# First-Person Virtual Embodiment Modulates the Cortical Network that Encodes the Bodily Self and Its Surrounding Space during the Experience of Domestic Violence

**DOI:** 10.1523/ENEURO.0263-19.2019

**Published:** 2020-05-20

**Authors:** Aline W. de Borst, Maria V. Sanchez-Vives, Mel Slater, Beatrice de Gelder

**Affiliations:** 1 University College London Interaction Centre, University College London, London WC1E 6EA, United Kingdom; 2 Institut d’Investigacions Biomèdiques August Pi i Sunyer (IDIBAPS), 08036 Barcelona, Spain; 3 ICREA, 08010 Barcelona, Spain; 4 Event Laboratory, Department of Clinical Psychology and Psychobiology, University of Barcelona, 08035 Barcelona, Spain; 5 Institute of Neurosciences of the University of Barcelona, 08035 Barcelona, Spain; 6 Brain and Emotion lab, Department of Cognitive Neuroscience, Faculty of Psychology and Neuroscience, Maastricht University, 6229 EV Maastricht, The Netherlands

**Keywords:** first-person perspective, fMRI, naturalistic neuroscience, peripersonal space, threat, virtual reality

## Abstract

Social aggression, such as domestic violence, has been associated with a reduced ability to take on others’ perspectives. In this naturalistic imaging study, we investigated whether training human participants to take on a first-person embodied perspective during the experience of domestic violence enhances the identification with the victim and elicits brain activity associated with the monitoring of the body and surrounding space and the experience of threat. We combined fMRI measurements with preceding virtual reality exposure from either first-person perspective (1PP) or third-person perspective (3PP) to manipulate whether the domestic abuse stimulus was perceived as directed to oneself or another. We found that 1PP exposure increased body ownership and identification with the virtual victim. Furthermore, when the stimulus was perceived as directed toward oneself, the brain network that encodes the bodily self and its surrounding space was more strongly synchronized across participants and connectivity increased from premotor cortex (PM) and intraparietal sulcus towards superior parietal lobe. Additionally, when the stimulus came near the body, brain activity in the amygdala (AMG) strongly synchronized across participants. Exposure to 3PP reduced synchronization of brain activity in the personal space network, increased modulation of visual areas and strengthened functional connectivity between PM, supramarginal gyrus and primary visual cortex. In conclusion, our results suggest that 1PP embodiment training enhances experience from the viewpoint of the virtual victim, which is accompanied by synchronization in the fronto-parietal network to predict actions toward the body and in the AMG to signal the proximity of the stimulus.

## Significance Statement

Using a combination of virtual reality and fMRI, our work reveals how first-person perspective (1PP) embodiment increases identification with the virtual victim during the experience of domestic abuse. We showed that when participants are embodied in the virtual victim the fronto-parietal brain network responsible for the representation of the bodily self and its surrounding space showed highly synchronized activity across participants when experiencing domestic abuse. Moreover, in this condition proximity of the aggressor strongly correlated with neural synchronization of the amygdala. We conclude that 1PP embodiment allows participants to identify with the virtual victim through changes in this fronto-parietal network.

## Introduction

Perspective taking enhances the ability to understand another person’s actions, thoughts and emotions. In cases where it is more difficult to visualize the other’s viewpoint specific training can be used to support perspective taking. In the virtual body ownership illusion, multisensory feedback, often combined with a first-person perspective (1PP), is used to create the illusion that a virtual body is part of the own body (Kokkinara and Slater, 2014). 1PP and the sense of ownership over one’s own body or an artificial body is supported by multisensory integration in the brain, where regions such as the ventral premotor cortex (vPM), intraparietal sulcus (IPS), primary somatosensory cortex (PSC), and temporoparietal cortex integrate information from different sensory modalities (Blanke, 2012; Ehrsson, 2012; Serino, 2019). These brain regions are thought to not only integrate information from the body, but also from the space directly surrounding the body: the peripersonal space (Rizzolatti et al., 1981). Research has shown that even when the virtual body has a different age, gender or race, the body ownership illusion leads people to take on the perspective and characteristics of the virtual character (Banakou et al., 2013; Hamilton-Giachritsis et al., 2018). This type of training has also been used in individuals with a reduced ability to take on others’ perspectives, such as violent offenders (Seinfeld et al., 2018). The peripersonal space can also be modulated by this illusion, such that actions in the space surrounding the artificial body are perceived as if they were close to the real body (Ehrsson et al., 2007). Here, we investigated how the brain allows for these changes in perspective, as this could form the basis for understanding how behavioral change in offenders could be supported through embodiment training. In specific, we studied whether taking on the viewpoint of a victim of domestic abuse increases activity in brain regions responsive to threat and protection of the body.

A vast body of literature has documented animal and human threat processing networks in the brain, which include subcortical structures, such as the amygdala (AMG), and the visuo-motor cortex for sensory detection and preparatory responses (LeDoux, 2003; Han et al., 2008). For fast decision-making on whether to defend against a potential threat it is vital to integrate information in nearby space across the different senses (Pereira and Moita, 2016). Recent animal studies have shown that different neural circuits mediate fear responses according to the nature and proximity of the threat (Silva et al., 2016). However, in humans threat has been mainly investigated with static images (Sussman et al., 2016; Fernandes et al., 2017), which contrasts with the relevance of movement in the perception of threat (Mobbs et al., 2010; Ahs et al., 2015). In this exploratory study, we used a naturalistic approach to investigate the neural correlates of perspective taking during threat. To this end we used virtual reality (VR), as this typically creates the perceptual illusion of “presence” and “plausibility” (Slater, 2009), which, together with 1PP and body ownership, typically lead participants to behave similarly in VR to how they would behave in reality (Banakou et al., 2013; Maister et al., 2015). The experimental design of this fMRI study follows earlier investigations using free viewing of natural scenes (Bartels and Zeki, 2004; Hasson et al., 2004), rather than repeated presentation of short static stimuli, to make the experience as realistic as possible. This approach does not allow for the use of conventional general linear model (GLM) analyses, as conditions are not repeated multiple times. Instead, we calculated the synchrony of brain activity across participants using intersubject correlation (ISC; Hasson et al., 2004), which allows for the analysis of brain responses without a-priori definition of the stimulus design. In order to control for all lower-level stimulus properties between the two conditions, we presented the participants with an identical 3D threat scenario during fMRI measurements in both conditions, with differences between conditions induced by a preceding 1PP or third-person perspective (3PP) VR exposure. We hypothesized that when the observer is primed to inhabit the space of the virtual victim information is transmitted from the visual and auditory regions to the multisensory integration areas in the vPM and IPS, where brain activity will be more strongly synchronized across participants. We also expected stronger synchronization of brain activity in threat processing regions such as the AMG, during 1PP experience of nearby threat, as the threat would be perceived to come close to the body.

## Materials and Methods

### Participants

Twenty healthy volunteers participated in this study. Half of the participants were male (mean age 22.3 years; range 19–28) and half were female (mean age 20.3 years; range 18–24). All participants had normal or corrected-to-normal vision and gave their informed consent. Exclusion criteria were the institute’s MRI safety criteria. Because of the nature of the stimuli, we also excluded volunteers who had a criminal record, or a history of physical or emotional abuse. The study was approved by the local ethical committee.

### Stimuli and materials

**Figure 1.**
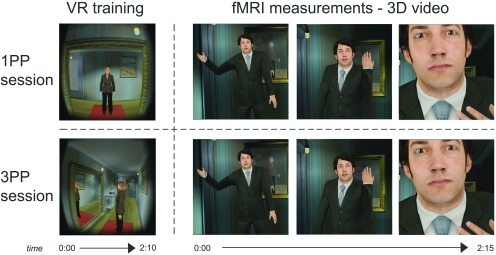
Experimental design. In two counterbalanced sessions, participants were immersed in a VR scenario (VR exposure) where they either observed the scenario from a 1PP and performed visuomotor exercises congruent with the female character’s movements (1PP; top left), or observed the scenario from a 3PP and performed visuomotor exercises congruent with the virtual camera’s movements (3PP; bottom left). In both sessions, participants were subsequently moved to an MRI scanner, where they watched a continuous 3D video from 1PP showing a domestic violence situation in which a male aggressor approached the viewer and entered their personal space (right).

The stimuli consisted of a VR scenario, two auditory stimuli containing instructions, and one 3D split-screen video. The VR scenario displayed a female avatar in the hallway of a house (Fig. 1, left). There were two mirrors in the hallway, two doors on opposite ends, and a sideboard. The scenario could be viewed from a 1PP, or a 3PP. The VR environment was built in Unity (Unity Technologies). The participants viewed the VR scenario using an Oculus Rift DK2 (Oculus VR), which is a head-mounted display especially designed to view VR. The Oculus Rift has an OLED display with a 960 × 1080 resolution per eye, and uses an infrared camera for positional tracking of the headset. Stereoscopic vision was obtained by projecting the stimulus in a slightly different angle to the left and right eye. Each of the auditory stimuli lasted 2:08 min and consisted of a female voice giving instructions for several visuomotor exercises in the VR scenario from either 1PP or 3PP (e.g., “move your head to the right until you see the edge of the mirror”). The 3D split-screen video shown in the MRI scanner was a recording of a VR domestic abuse scenario from 1PP (Fig. 1, right). In this abuse scenario, a male avatar entered the hallway from one of the doors and started addressing the female avatar in a demeaning and aggressive manner. Over the course of 2:37 min, the male avatar throws the phone through the hallway and approaches the female closely while continuing to verbally abuse her. The 3D video was viewed inside the MRI scanner using VisStim MRI-compatible goggles (Resonance Technology). The VisStim goggles contain two displays, each with a 600 × 800 resolution, set within a rubber head mount. Similar to the Oculus Rift, stereoscopic vision was obtained by projecting the split-screen video onto the two screens. A VR questionnaire consisting of 17 items relating to different aspects of the VR experience was used to assess embodiment.

### Experimental procedure

The experiment consisted of two different sessions, which were one week apart. In the first session, participants were informed about the study and signed the informed consent form. Afterwards, the participants were familiarized with the MRI environment. Subsequently, outside of the scanner room, they put on the Oculus Rift and followed auditory instructions to perform several visuomotor exercises. They saw the VR scenario from either the 1PP or the 3PP (counterbalanced). In the 1PP session they looked into a full-length mirror and saw the female avatar performing head movements that were consistent with the participants’ own movements. The synchrony between the female avatars’ movements and the participants’ own movements contributes to the illusion of body ownership (Banakou and Slater, 2014; Fig. 1, top left). In the 3PP session, the participants performed the same movements as indicated by the auditory instructions, but instead they viewed the mirror and female avatar from a slight distance. In this perspective, the virtual camera viewpoint, rather than the female avatar, moved consistently with the participants’ head movements. The participants did not have a virtual body and therefore the visuomotor exercises could not contribute to the body ownership illusion (Fig. 1, bottom left). During the VR exercises the participants were not exposed to the virtual threat. After the first-person or third-person visuomotor exercises with the Oculus, the participants were blindfolded (to maintain the 1PP or 3PP illusion) and led to the magnetic resonance imaging (MRI) scanner. During functional MRI (fMRI) measurements, the participants experienced a 3D video of the VR environment where they, from the perspective of the female character, were verbally and psychologically abused by an approaching male character (Fig. 1, right). The 3D video shown during fMRI measurements was identical in both sessions. After the fMRI measurements participants filled out the VR questionnaire. At the end of the first session, participants were partially debriefed about the study, were asked about how they experienced the scenario and if they were affected by it. Moreover, they were asked to contact the experimenter if they had any reoccurring thoughts or feelings about the experiment.

After a week, participants came back to the laboratory and followed the same procedure as during the first session, but during this second session they performed the VR exercises from the other perspective (e.g., if they viewed it from 3PP in the first session, then they viewed it from the 1PP in the second session). All other aspects of the session were identical. They also filled out the VR questionnaire again at the end of the session and were debriefed about the contents and meaning of the study. Again, the emotional state of the participants was assessed and they were asked to contact the experimenter if they had any reoccurring thoughts or feelings, or were otherwise affected by participating in this experiment. No participant reported to be distressed by the experiment or have persisting thoughts or feelings about the experiment.

### Design

The order of the VR exposure perspective (1PP vs 3PP) between sessions was counterbalanced across participants, so that half of the males and half of the females had the session order 1PP-3PP and the other halves had the opposite order. The 3D video that was watched during fMRI measurements was identical in both sessions and was preceded and followed by 3 s of fixation. The two experimental conditions were the perception of the 3D video preceded by 1PP VR exposure (1PP session) and the perception of the 3D video preceded by 3PP VR exposure (3PP session). The 3D stimulus was shown once in each session, similar to other naturalistic research (Hasson et al., 2004).

### Data acquisition

A 3T Siemens MR scanner (MAGNETOM Prisma, Siemens Medical Systems) with a 64-channel head/neck coil was used for imaging. Functional scans were acquired with a multiband gradient echo echo-planar imaging sequence with a repetition time (TR) of 1500 ms and an echo time (TE) of 30 ms. The functional run consisted of 90 volumes comprising 57 slices (matrix = 100 × 100, 2-mm isotropic voxels, inter slice time = 26 ms, flip angle = 77°, multiband factor = 3, iPAT = 2). After the functional run, high-resolution T1-weighted structural images of the whole brain were acquired with an MPRAGE with a TR of 2250 ms and a TE of 2.21 ms, 192 slices (matrix = 256 × 256, 1 mm isotropic voxels, flip angle = 9°).

### Statistical analyses

#### Questionnaire analyses

The VR questionnaire contained questions relating to the subjective experience of the 3D domestic violence scenario. The scores on the VR questionnaire were compared between sessions (1PP vs 3PP exposure) by conducting an Wilcoxon signed-rank test (two-tailed) on all items, corrected for multiple comparisons using a false discovery rate (FDR) of 0.05 (Benjamini and Hochberg, 1995). All significant results are visualized in the results section. From the VR experience questionnaire, we used the scores on the question “To what extent did you feel in the female body and lived the situation as if you were the woman?”, the question “To what extent did you feel identified with the female body during the experience?” and the question “To what extent have you experienced the situation as if it was real?” to analyze, respectively, the perceived body ownership, identification and plausibility during the perception of the domestic violence scenario.

#### Functional MRI pre-processing

The fMRI data were pre-processed and visualized using fMRI analysis and visualization software BrainVoyager QX version 2.8.4 (Brain Innovation B.V.). Functional data were corrected for head motion (3D motion correction, sinc interpolation), corrected for slice scan time differences and temporally filtered (high pass, GLM-Fourier, 2 sines/cosines). For the ISC analyses, it is recommended to spatially smooth the functional data with a Gaussian smoothing kernel of slightly larger than double the original voxel size (Pajula and Tohka, 2014). Therefore, the functional data were spatially smoothed using a Gaussian kernel with a full-width at half-maximal of 5 mm. The anatomic data were corrected for intensity inhomogeneity (Goebel et al., 2006) and transformed into Talairach space (Talairach and Tournoux, 1988). The functional data were then aligned with the anatomic data and transformed into the same space to create 4D volume time courses (VTCs).

Head motion was below 1.1 mm for every participant (voxel size = 2 mm). We calculated the frame-wise displacement (FWD; Power et al., 2012) for every participant in each session and compared the FWD between sessions using a paired *t* test, corrected for multiple comparisons using a FDR of 0.05, to ensure that head motion did not differ between sessions. We found no differences in FWD between conditions at any time point (FDR > 0.8). Out of all runs of the 1PP session (20 participants × one session) 90% of runs had 98.9% of time points with a FWD under 0.5 mm (Power et al., 2012). Out of all runs of the 3PP session (20 participants × one session) also 90% of runs had 98.9% of time points with a FWD under 0.5 mm. We also calculated the mean FWD across time points for each participant and compared this between sessions using a paired *t* test. We found no difference between sessions for the mean FWD (*t*_(19)_ = −0.1212, *p* = 0.9048). For all participants and sessions, the mean FWD was below 0.5 mm (maximum = 0.2 mm). The grand mean FWD (mean across participants and time points) was 0.0962 for the 1PP session and 0.0958 for the 3PP session.

#### Anatomical mask

For the connectivity analyses and the second set of ISC analyses (see below), we defined anatomic regions of interest (ROIs), which included regions relevant for perspective taking, embodiment, peripersonal space, and emotion processing, as well as the superior parietal lobe (SPL), primary auditory cortex (PAC), the primary visual cortex (PVC) and middle temporal area (MT/V5) (Table 1). Statistical parametric mapping (SPM; version 12, Functional Imaging Laboratory) was used to extract probabilistic cytoarchitectonic maps from the SPM Anatomy Toolbox (version 1.8, Forschungszentrum Jülich GmbH; Eickhoff et al., 2005). For regions that were not included in the Anatomy Toolbox we used the Hammers Adult maximum probability atlases (Hammers et al., 2003).

**Table 1:** Mean Talairach coordinates and cluster sizes of the ROIs that were defined on the basis of anatomic probability atlases and used in the ISC and connectivity analyses

Name	Hemisphere	Cluster size	Mean Talairach coordinates		
			*x*	*y*	*z*
PM	Left	91529	–30	–12	44
	Right	91242	28	–15	44
IPS	Left	38404	–35	–52	38
	Right	40746	36	–53	39
PSC	Left	85135	–32	–26	43
	Right	93873	31	–29	43
SMG	Left	68995	–44	–37	33
	Right	70282	42	–39	32
PVC	Left	36337	–13	–81	1
	Right	35727	14	–77	2
PAC	Left	13410	–45	–20	11
	Right	10218	46	–17	10
MT/V5	Left	6530	–41	–69	2
	Right	6685	46	–64	0
SPL	Left	78210	–18	–58	46
	Right	69199	18	–57	48
AMG	Left	5169	–22	–4	–13
	Right	5326	21	–5	–13
ACC	Left	23304	–7	20	31
	Right	28025	5	21	31
aINS	Left	16817	–31	8	11
	Right	17361	29	8	10

Each voxel in a probabilistic region reflects the cytoarchitectonic probability (10–100%) of belonging to that region. We followed a procedure to obtain maximum probability maps as described in Eickhoff et al. (2006), as these are thought to provide ROIs that best reflect the anatomic hypotheses. This meant that all voxels in the ROI that were assigned to a certain area were set to “1” and the rest of the voxels were set to “0.” The ROIs were transformed from MNI space to Talairach space (as Talairach space was used in the other analyses). We extracted the Colin27 anatomic data to help verify the subsequent transformations. In order to transform the ROIs and the anatomic data from MNI space to Talairach space, we imported the ANALYZE files in BrainVoyager, flipped the *x*-axis to set the data to radiological format, and rotated the data −90° in the *x*-axis and +90° in the *y*-axis to get a sagittal orientation. Subsequently, we transformed the Colin27 anatomic data to Talairach space (Talairach and Tournoux, 1988; Goebel et al., 2006) and applied the same transformations to the cytoarchitectonic ROIs. For the ISC time course analysis on emotion processing (see section below), we used the bilateral probabilistic cytoarchitectonic maps of the AMG, anterior cingulate cortex (ACC) and the anterior subdivision of the insula (aINS) as ROIs (excluding the middle and posterior subdivisions of the insula).

#### ISC

The ISC toolbox for fMRI in MATLAB (Kauppi et al., 2014) and in-house MATLAB scripts were used for ISC analyses with MATLAB version R2013b, 8.2.0.701 (The MathWorks Inc.). We calculated group-level ISC maps and ISC difference maps to reveal significant differences between conditions as described in Kauppi et al. (2014). Pearson’s correlation coefficient was used to calculate voxel-wise temporal correlations between the VTCs (90 volumes) of all possible subject pairs (*N* (*N* – 1)/2). The group-level ISC is the sum of these correlation coefficients divided by the number of subject pairs. The ISC maps for each condition were corrected using an FDR of 0.001. The ISC difference maps between the sessions were calculated as described in Kauppi et al. (2014, their section 2.2.4). In the ISC difference analyses a Fisher z-transformation (ZPF; Fisher, 1915) was applied to each pairwise correlation in each voxel. Subsequently, a sum ZPF statistic for the difference between the two conditions (1PP and 3PP) was calculated over all subject pairs and tested against the null hypothesis that each ZPF value comes from a distribution with zero mean (no difference between 1PP and 3PP) using non-parametric permutation testing (as the stimuli in the two conditions may not be independent). The null distribution was obtained by randomly flipping the sign of pairwise ZPF statistics before calculating the sum ZPF statistic using 25,000 permutations (for more details, see Kauppi et al., 2014). Maximal and minimal statistics over the entire image corresponding to each labeling were saved. The map was thresholded at α = 0.05 using the permutation distribution of maximal statistic, which accounts for the multiple comparisons problem by controlling the FWER (Nichols and Holmes, 2002).

**Figure 2.**
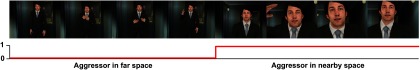
Visualization of the threat proximity predictor.

In a second set of analyses, we correlated the time course of the ISC in emotion processing regions with a predictor that coded for aggressor proximity to understand whether emotion-related brain activity becomes more synchronized across participants with approaching threat. In order to obtain the time course of the ISC in each condition we calculated the average ISC in eight non-overlapping time windows in three emotion-relevant ROIs (AMG, ACC, aINS). The first two volumes of the time courses were excluded and the remaining 88 volumes were separated in eight time windows of 10 volumes, which were each separate by 1 volume. Although short time windows could result in less reliable estimates, the minimal window length depends on the stimulus and sample size. Windows of 10 samples have been used previously with 16 participants (Nummenmaa et al., 2012). Our approach resulted in group-level ISC maps for each of the eight time windows per condition and ROI, reflecting the moment-to-moment degree of intersubject synchronization across participants (Kauppi et al., 2014, their section 2.2.1). Subsequently, the voxel-wise correlation coefficients for all possible subject pairs were transformed to *z* scores using a Fisher z transformation and the mean across all subject pairs was calculated by summing the transformed correlation coefficients and dividing by the number of subject pairs (Kauppi et al., 2014, their section 2.2.3). The mean across all voxels of an ROI was calculated for each of the eight maps, resulting in a mean time course of ISC with eight time points (one per time window) for each ROI and condition. The resulting time courses were correlated with a predictor that coded for the position of the aggressor in the 3D space during the course of the scenario. This predictor consisted of a box-car function with “0” for the first four time windows that corresponded to the time during which the aggressor was in far space (∼2-m distance), and “1” for the remaining four time windows that corresponded to the time during which the aggressor was in nearby space (∼30-cm distance; Fig. 2). The correlation coefficients were tested against the null hypothesis that there is no relationship between the observed phenomena (df = 6). The results were corrected for multiple comparisons using an FDR of 0.05.

#### Connectivity analyses

We used the RFX Granger Causality Mapping (GCM) plugin of BrainVoyager QX 2.8 and in-house MATLAB scripts to calculate effective connectivity between brain regions. GCM (Roebroeck et al., 2005) uses vector autoregressive models of fMRI time-series in the context of Granger causality. A time-series of voxel X Granger causes a time-series of voxel Y, if the past of X improves the prediction of the current value of Y, given that all other relevant sources of influence have been taken into account (including the past of Y). As we had few hypotheses about the directions of connectivity between regions, we choose to use GCM because it does not require a-priori modeling of the connectivity. Given the large amount of regions included in our network, it did not seem feasible to map all possible connections, which would be required for other approaches, such as dynamic causal modeling. We obtained whole-brain maps of directed influence (dGCM) per session for each of the ROIs (Table 1) in each individual participant (*N* = 20), i.e., each region served as a reference region. These dGCM maps are maps with an influence difference term for each voxel that has a positive value where influence from the reference region dominates and a negative value where influence to the reference region dominates (Roebroeck et al., 2005). The individual dGCM maps were corrected for multiple comparisons using an FDR of 0.01. For each reference region the 40 individual dGCM maps (20 participants, two sessions) with the influence difference statistics were subsequently used in a second level RFX group analysis to calculate differences in directed connectivity between conditions. For the group analysis a RFX ANOVA, with condition (1PP vs 3PP) as a within-subjects factor, was calculated for each reference region. The resulting whole-brain F-maps were corrected for multiple comparisons using the cluster level statistical threshold estimator plugin of BrainVoyager QX 2.8 (Goebel et al., 2006) with an initial threshold of *p* = 0.005 and a cluster size corrected threshold of *p* < 0.05. In order to counteract a potential downfall of GCM that directed connections may reflect inherent hemodynamic differences between regions (Deshpande et al., 2010), we compared effective connectivity between two conditions in an identical set of regions. If there are inherent hemodynamic differences between regions, which give rise to false connections, these would be present in both conditions. By only taking into account differences in connections between conditions we circumvent the above-mentioned problem.

In a second set of analyses, we calculated the functional connectivity between regions for both conditions using instantaneous GCM (correlation). Functional connectivity between two time-series X and Y exists when the values of time-series X enhance predictions of contemporaneous values of the time-series Y, taking into account other sources of influence (past X and Y; Roebroeck et al., 2005). Similar to the first set of dGCM analyses, we obtained whole-brain maps of instantaneous correlations per session for each of the ROIs (Table 1) in each individual participant (*N* = 20), i.e., each region served as a reference region. For each reference region the 40 individual, instantaneous GCM maps (20 participants, two sessions) were subsequently used in a second level RFX group analysis to calculate differences in functional connectivity between conditions. For the group analysis, a RFX ANOVA, with condition (1PP vs 3PP) as a within-subjects factor, was calculated for each reference region. The resulting whole-brain F-maps were corrected for multiple comparisons using the cluster level statistical threshold estimator plugin of BrainVoyager QX 2.8 (Goebel et al., 2006) with an initial threshold of *p* = 0.005 and a cluster size corrected threshold of *p* < 0.05.

## Results

### Subjective experience of perspective

**Figure 3.**
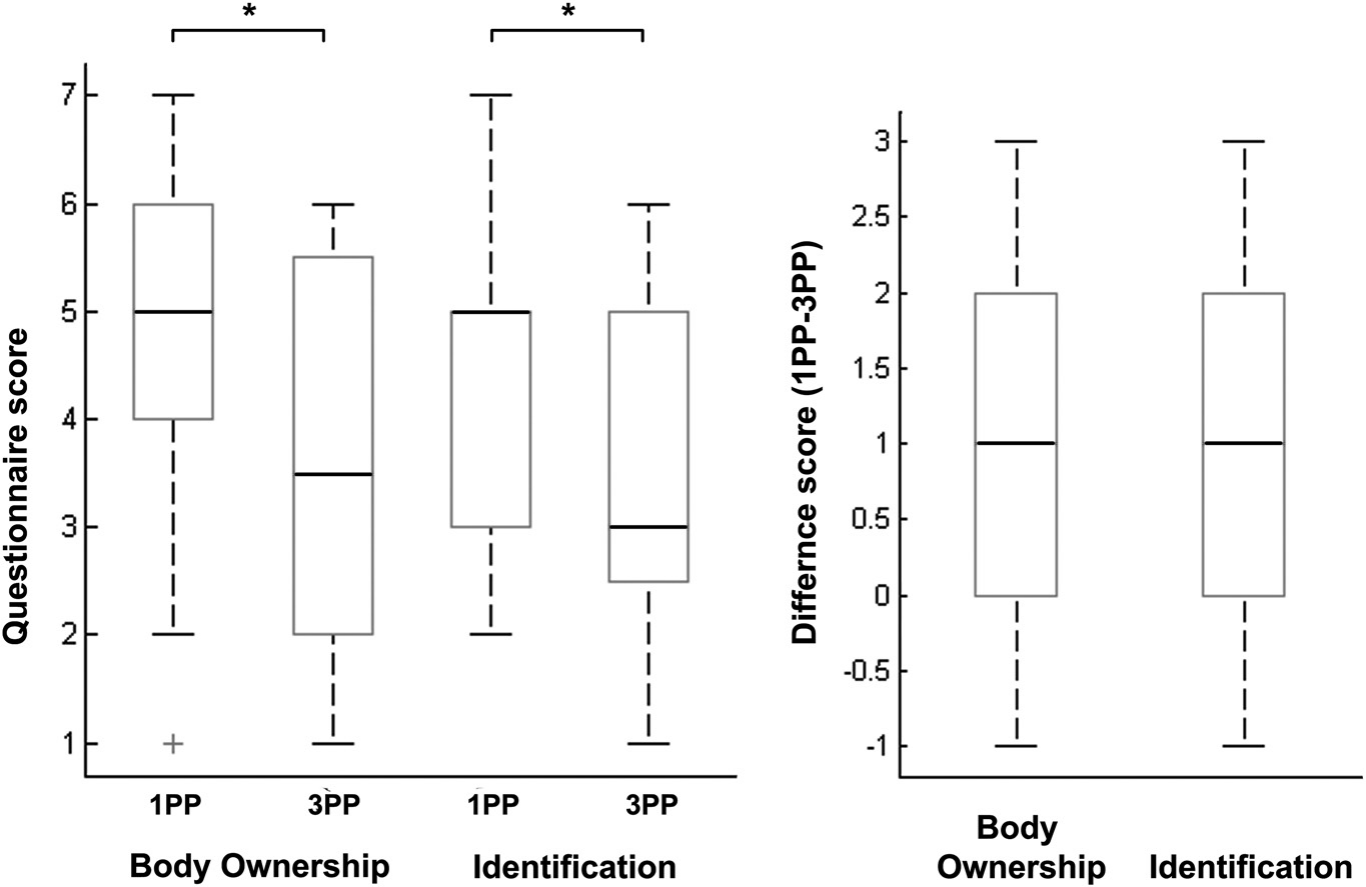
Boxplots of VR questionnaire data. Left, The boxplots show the medians, interquartile ranges, maximum and minimum (as indicated by the stems) and outliers of the questionnaire scores that addressed the subjective experience of body ownership and identification for each condition (1PP, 3PP). An asterisk indicates a significant difference (FDR < 0.05). Right, The boxplots show the medians, interquartile ranges, maximum and minimum of the difference scores (1PP-3PP) for body ownership and identification.

We assessed how participants experienced the VR scenario by analyzing the VR questionnaires that were administrated at the end of each session. All questions were scored on a 1 (not at all) to 7 (completely) Likert scale. The differences in answer scores between 1PP and 3PP conditions were analyzed using Wilcoxon signed-rank test, corrected for multiple comparisons at FDR = 0.05 (see Materials and Methods). Our main questions of interest related to differences in perceived body ownership, identification and plausibility. We found that participants reported higher body ownership and identification during the VR threat scenario in the MRI scanner (Fig. 3) when they were previously exposed to 1PP combined with visuo-motor exercises that were synchronous with the virtual body than when they were exposed to 3PP combined with visuo-motor exercises without a virtual body.

Identification was assessed with question 1: “To what extent did you feel identified with the female body during the experience?”. The results of the Wilcoxon signed-rank test showed a significant difference between conditions (FDR = 0.03), with higher scores of identification in the 1PP session (1PP: 4.45 ± 0.33; 3PP: 3.50 ± 0.30; Fig. 3). Body ownership was assessed with question 2: “To what extent did you feel in the female body and lived the situation as if you were the woman?”. The results of the Wilcoxon signed-rank test showed a significant difference between conditions (FDR = 0.04), with higher scores of body ownership in the 1PP session (1PP: 4.85 ± 0.37; 3PP: 3.75 ± 0.39; Fig. 3). Plausibility was assessed with question 3: “To what extent have you experienced the situation as if it was real?”. The results of the Wilcoxon signed-rank test did not show a significant difference between sessions, but did show a trend for higher scores in the 1PP session compared with the 3PP session (1PP: 4.70 ± 0.36; 3PP: 3.70 ± 0.32; FDR = 0.05). No other item of the VR questionnaire showed a significant difference between the conditions. Two items showed a trend for higher scores in the 1PP session compared with the 3PP session. This was question 12.3: “To what extent did you find the following behaviors of the male avatar threateing – When he invades your peripersonal space and moves his hands?” (1PP: 5.75 ± 0.31; 3PP: 4.90 ± 0.29; FDR = 0.05) and question 15: “Did you feel that the virtual man was speaking to and addressing you personally?” (1PP: 4.05 ± 0.36; 3PP: 3.30 ± 0.33; FDR = 0.05).

### fMRI approach

In this study we wanted to explore perspective taking in the context of social threat in a naturalistic manner. Therefore, we prioritized having two sessions with one continuous stimulus rather than a more conventional approach with repeated presentation of different stimulus conditions in one session. The nature of our complex stimulus meant that we could not use conventional analysis methods, such as the GLM. Instead, we used ISC (Hasson et al., 2004) to compare spatiotemporal activity across participants during the course of the natural stimulus perception. In this manner, we could identify stimulus-locked neural responses across brains. In ISC analysis, the shared neural processing of participants is defined by calculating the correlation coefficient between fMRI time-series of participants in locations across the brain (see Materials and Methods). This makes ISC particularly suitable for naturalistic stimuli, such as 3D video, as it does not require modeling of the stimuli (Hasson et al., 2004, 2008; Nummenmaa et al., 2012). After calculating an ISC map for each condition, we calculated ISC difference maps, which show the statistical difference between conditions in each voxel.

### Fronto-parietal network involved during 1PP induced threat perception

**Figure 4.**
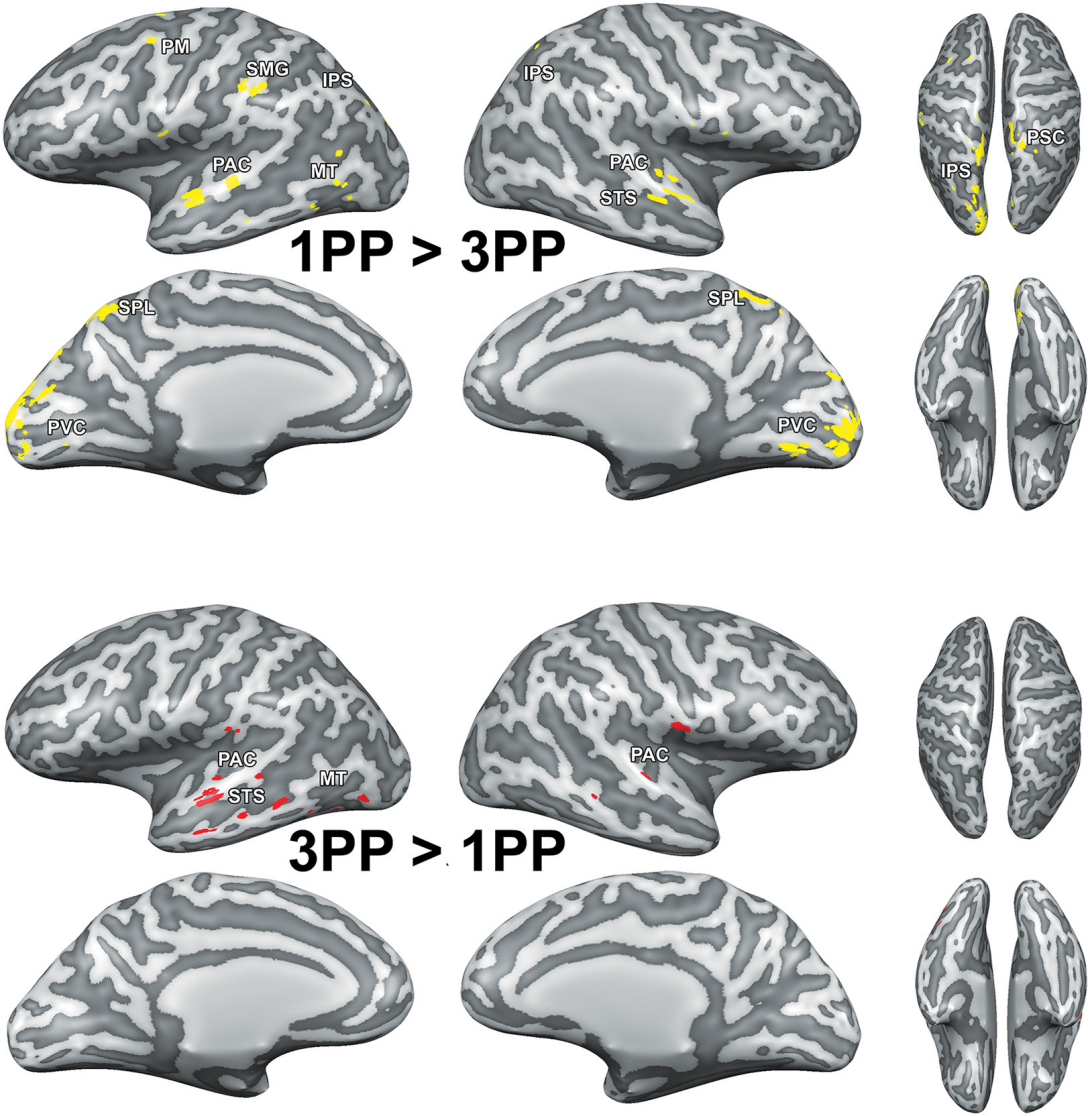
ISC differences (permutation testing, *N* = 20, *p*[corrected] < 0.05) between perception of an identical 3D domestic violence video preceded by 1PP and 3PP exposure. Voxels that showed significantly higher ISC after 1PP exposure than 3PP exposure are indicated in yellow (top). Voxels that showed significantly higher ISC after 3PP exposure than 1PP exposure are indicated in red (bottom).

The results of the fMRI analyses confirmed our first hypothesis: the multisensory network related to 1PP, body ownership and peripersonal space representation was more strongly synchronized across participants after first than after 3PP exposure. The ISC difference maps (*N* = 20, *p*[FWER] < 0.05) showed that 1PP exposure induced higher ISC than 3PP exposure in left dorsal and ventral PM, in bilateral IPS, left SMG, bilateral SPL, right PSC and bilateral PVC during perception of the 3D video (Fig. 4, top). Additionally, after both 1PP (Fig. 4, top) and 3PP exposure (Fig. 4, bottom) differences in ISC were found in different areas of the bilateral PAC, superior temporal sulcus (STS) and left MT.

### VR exposure influences effective connectivity in the fronto-parietal network

**Figure 5.**
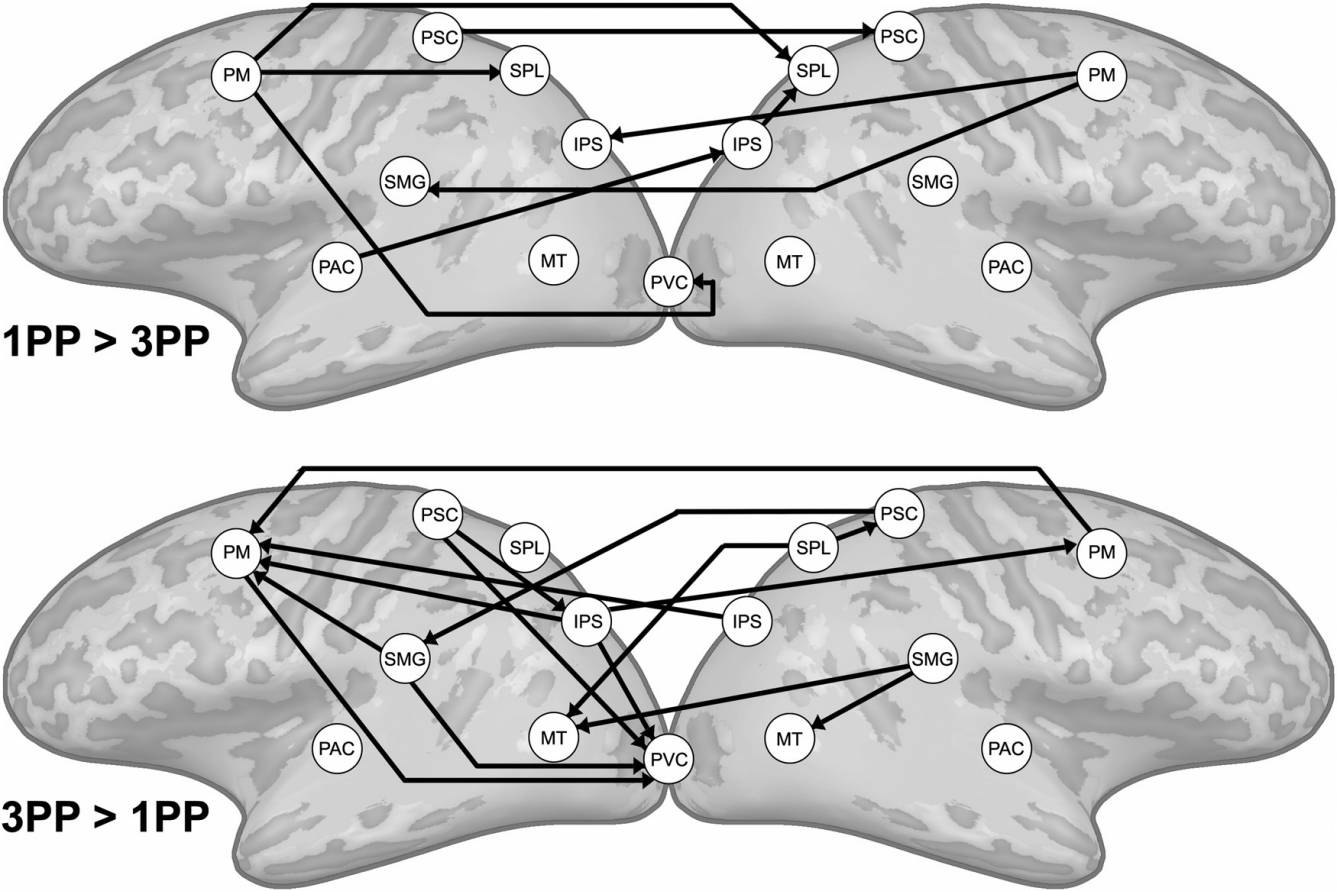
Differences in effective connectivity between perception of an identical 3D threat video preceded by 1PP and 3PP exposure (RFX ANCOVA, *N* = 20, *p*[corrected] < 0.05). The arrows indicate the direction of the connectivity between regions that is unique for each condition.

**Figure 6.**
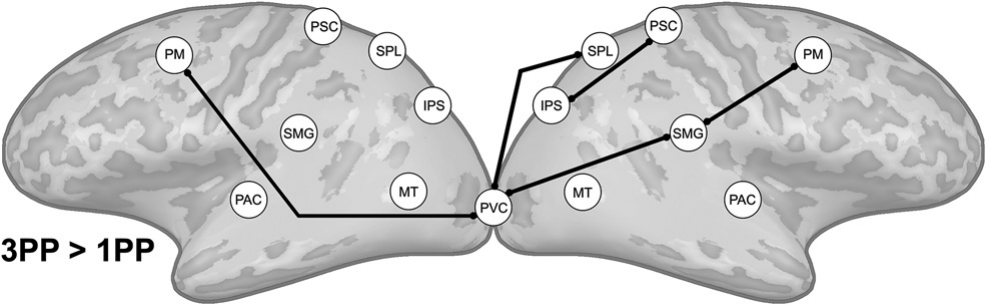
Differences in functional connectivity between perception of an identical 3D threat video preceded by 1PP and 3PP exposure (RFX ANCOVA, *N* = 20, *p*[corrected] < 0.05).

The results from the effective connectivity analyses did not confirm our second hypothesis, that visual and somatosensory regions would send information to PM and IPS during first-person embodied threat perception. We calculated effective connectivity differences between the two conditions in an identical set of regions using RFX ANCOVA analyses (see Materials and Methods, *N* = 20, *p*[corrected] < 0.05). We found that during threat perception preceded by 1PP exposure directed connections from PM, IPS, SMG, and MT toward SPL were stronger (Fig. 5, top). This appears to suggest that information was integrated in SPL. These findings are in line with the ISC results, which also emphasized changes in these regions during the 1PP session. Moreover, we found that bilateral PM showed directed connections to many of the other regions in the network. Additionally, not shown in Figure 5, we found stronger directed connections from left PAC and left IPS to right ACC and from right ACC to right AMG and left ACC after 1PP exposure. Although we found that ISC was reduced in the fronto-parietal network during threat perception in the 3PP session, the connectivity results revealed a more complex situation (Fig. 5, bottom). Contrary to the 1PP session, we found stronger directed connectivity from IPS to PM, but information did not converge in posterior parietal cortex. Moreover, we found enhanced top-down connectivity from PM, IPS, SMG, SPL and PSC toward visual areas PVC and MT. Similarly, we also found that functional connectivity increased between PM, SMG, and SPL and the PVC in the 3PP session (Fig. 6). Additionally, not shown in Figure 5, we found a directed connection from left SPL to right ACC after 3PP exposure.

### Effect of first-person perspective on threat processing in nearby space

**Figure 7.**
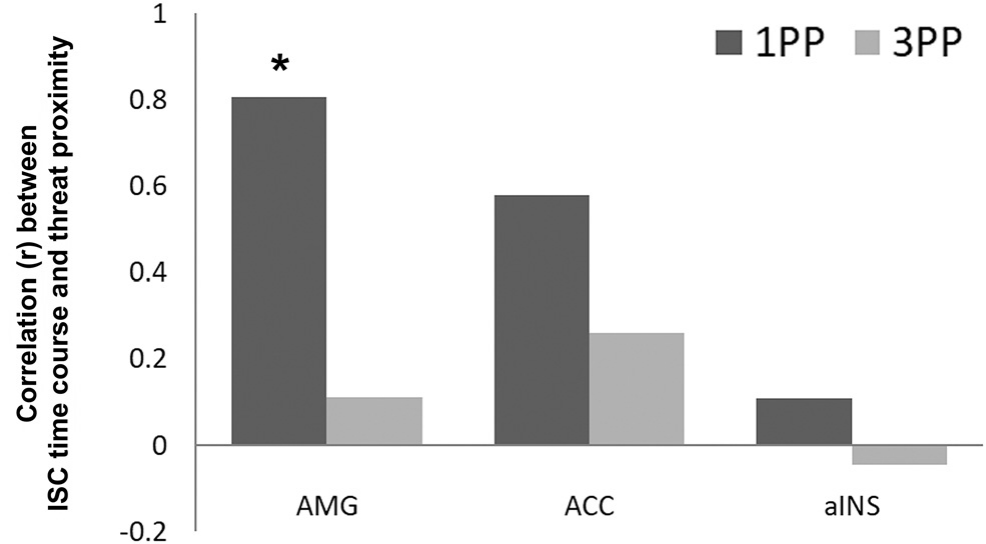
Correlation between threat proximity and the ISC time course within each ROI in the 1PP (dark gray) and the 3PP (light gray) session. An asterisk indicates a significant relationship between threat proximity and ISC (FDR < 0.05).

Finally, we investigated whether activity in emotion-related structures, such as AMG, is more strongly synchronized across participants when the participants had 1PP embodied exposure than 3PP exposure before the experience of nearby threat. Although we found no evidence for explicit experience of increased threat in the 1PP session in the questionnaire data, the trend (FDR = 0.05) for higher scores after 1PP exposure found on question 12.3: “To what extent did you find the following behaviors of the male avatar threatening – when he invades your peripersonal space and moves his hands?”, suggests that the intrusion into the personal space after 1PP exposure may have had an effect on the consciously or subconsciously perceived threat. We calculated the time course of ISC in the ROIs of three emotion-related structures (bilateral AMG, ACC, and aINS) using eight non-overlapping time windows of 10 volumes (see Materials and Methods). Subsequently, we correlated the time course of the ROI with a predictor that coded for threat proximity. We found (Fig. 7) that during the 1PP session, the time course of ISC in the AMG significantly correlated with threat proximity (*R* = 0.8062, FDR < 0.05), while it did not in the 3PP session (*R* = 0.1107, FDR > 0.9). For the ACC and the aINS, the ISC time courses did not correlate significantly with threat distance in the 1PP session (ACC: *R* = 0.5796, FDR > 0.1; aINS: *R* = 0.1078, FDR > 0.7) and the 3PP session (ACC: *R* = 0.2607, FDR > 0.9; aINS: *R* = −0.0443, FDR > 0.9). These results indicate that when the participants were embodied in the virtual victim after 1PP exposure, the increased intersubject synchronization of the AMG signaled the proximity of the aggressor, while this was not the case after 3PP exposure.

## Discussion

### Influence of perspective exposure on fronto-parietal network

Our first hypothesis was that synchronization of brain activity across participants in the fronto-parietal network will be stronger when a threat is perceived to be in our own space (1PP embodied exposure) than in another’s space (3PP exposure). The results of the ISC analyses indicated a clear effect of 1PP versus 3PP exposure on the neural synchronization in this network. After 1PP exposure we found that all regions related to body ownership and nearby space representation, including PM, IPS, SMG, SPL, and PSC, were more synchronized across participants. This was not the case when participants were exposed to 3PP. We expected that during threat perception in the 1PP session sensory information from PVC, PAC, and PSC would converge via the IPS in the PM, as the PM should initiate the defensive responses. We based this hypothesis on the fact that electrical stimulation of F4 and VIP in monkeys (human homologues of superior vPM and ventral IPS) produces movements similar to defensive movements followed by air puffs (Cooke and Graziano, 2003, 2004) and on the existing anatomic connections from monkey VIP to F4 (Luppino et al., 1999). Research in humans also indicated the involvement of PM in threat-related processing (Pichon et al., 2012). Therefore, the ventral PM-IPS network is believed to function to protect the near personal space (Cléry et al., 2015). Our findings do indicate the involvement of superior ventral PM and IPS (Fig. 4), but show a convergence of information in SPL rather than vPM in the 1PP session. The SPL is an area where many different cognitive functions converge, including attention (Kastner et al., 1999), spatial imagery and perception (Ungerleider and Haxby, 1994; Formisano et al., 2002; de Borst et al., 2012), and the generation and guidance of actions (Culham and Kanwisher, 2001). The superior parietal cortex also underlies the transformation of multisensory input to different coordinate systems, e.g., head, arm, body centered (Andersen et al., 1993), and converting this information into motor commands and whole body actions to targets (Buneo and Andersen, 2006; Whitlock et al., 2008). Together with the PM and IPS, the SPL may monitor, predict and evade intrusive actions toward the body (Lloyd et al., 2006; Cléry et al., 2015). Our results indicate that these brain regions synchronize more strongly across participants when participants are embodied in the virtual victim of domestic abuse.

After 3PP exposure, we observed that brain activity in this fronto-parietal network was not synchronized consistently across participants. We hypothesize that this may indicate that after first-person embodiment the body and nearby space representation is aligned with the virtual body and intrusion of this space synchronizes activity across participants in the fronto-parietal network, while after 3PP exposure this does not occur. Indeed, our ISC findings suggest that exposure to a 3PP does not synchronize the personal space network and this aspect of the results did not provide support for a shared peripersonal space that is independent of perspective. However, the effective connectivity results did show 3PP specific modulations in the fronto-parietal network. We found an increase in connectivity from IPS to PM and an increase of top-down connectivity from the peripersonal space network nodes (PM, IPS, SMG, PSC) toward the PVC and MT. The former results indicate that although information was sent from IPS to PM in line with peripersonal space encoding models and existing anatomic connections (Luppino et al., 1999), the ISC results showed that the underlying brain activity in these regions was not very similar across participants. Nevertheless, these results do indicate a role for peripersonal space representation even if the stimulus is perceived passively in the victim’s space, perhaps for the protection of others. Moreover, we found that after 3PP exposure there was overall more top-down connectivity to visual areas. The increased effective and functional connectivity between PVC and IPS, SPL, and SMG suggests that monitoring of the moving visual stimulus took place after 3PP exposure. Although the video that the participants viewed in the MRI scanner was identical in both conditions, the top-down modulation of visual areas we observed here may indicate that the imposed 3PP altered spatial attention, similar to how task differences can alter attention during identical stimulation (Li et al., 2004).

### Role of the temporoparietal cortex

Previous research has shown that the temporoparietal cortex [including the temporoparietal junction (TPJ) and SMG] is implicated in perspective taking (Maguire et al., 1998; Ruby and Decety, 2001; Falk et al., 2012; Besharati et al., 2016), interoception (Kashkouli Nejad et al., 2015), and self-other distinction (Steinbeis et al., 2015). For example, viewing painful stimulation of a hand evoked activity in bilateral SMG, which was linked to simulated pain to the own body (Costantini et al., 2008). The TPJ/SMG has also been mentioned as part of the multisensory representation of peripersonal space (Lloyd et al., 2003; Makin et al., 2007; Brozzoli et al., 2012; Grivaz et al., 2017). The results of our ISC analyses indicated that the SMG is more strongly synchronized during threat perception preceded by 1PP than 3PP exposure. However, the connectivity analyses indicate that the SMG was an integrated part of the network in both conditions. In the 1PP session, it received information from PM and sent information to SPL, which is in line with anatomic connections between the SMG, ventral PM and IPS in the human brain (Rushworth et al., 2006). In the 3PP session, the SMG sent information to visual areas and PM and was functionally connected with these regions as well. These results suggest that the temporo-parietal cortex plays a more general role in perspective taking and relating body relevant information to the self.

### Affective responses to first-person experience of threat

Our second hypothesis focused on synchronized brain activity in emotion-processing regions during 1PP experience of nearby threat. Threat monitoring and defensive responses are especially enhanced when the threat is near, as shown by a series of experiments with virtual characters (Ahs et al., 2015) and threatening stimuli (Mobbs et al., 2009, 2010; de Haan et al., 2016; Wabnegger et al., 2016). A recent study showed that the AMG is more activated when moving from threat anticipation to threat confrontation (Klumpers et al., 2017). Our results support these findings. We found that brain activity is more synchronized across participants in the AMG when the stimulus was perceived to be near the body (1PP condition). We found this effect only in the AMG, not in other emotion-relevant structures such as the ACC and aINS. This further emphasizes the special role of the AMG in regulating interpersonal distance by signaling when stimuli approach the body (Kennedy et al., 2009; Klumpers et al., 2017). We found no evidence for enhanced ISC in any of the emotion processing regions when the stimulus was perceived as directed to another person. The findings of the subjective experience questionnaire indicated that the VR exposure from 1PP was effective in eliciting identification with the virtual victim and thereby may have enhanced synchronization of (non-conscious) affective responses to nearby threat (in line with Galvan Debarba et al., 2017). Although we found no direct evidence for increased experience of threat after 1PP exposure, previous research has shown that emotion processing often takes place on a non-conscious level (Tamietto and de Gelder, 2010). We did find a trend for higher scores on the experience of threat when the aggressor entered the peripersonal space after 1PP exposure (see Results), which is in line with the increased synchronization of brain activity across participants in the AMG during this event.

In addition, we found modulations of connectivity toward the emotion processing regions during the perception of the scenario, both after 1PP and 3PP exposure. The connectivity analyses revealed that in the 1PP session PAC and IPS sent information to right ACC and from the right ACC information was forwarded to right AMG and left ACC. The ACC has a general role in decision-making and social and emotional processing (Lavin et al., 2013) and has bilateral connections to the PM and auditory cortex (Jacobson and Trojanowski, 1977; Müller-Preuss et al., 1980; Vogt and Pandya, 1987; Barbas, 1988; Petrides and Pandya, 1988; Barbas et al., 1999). Moreover, the ACC is linked to vocalizations (Müller-Preuss and Ploog, 1981; Dolan et al., 1995; Frith and Dolan, 1996) and auditory processing of speech in humans (Paus et al., 1993; Frith et al., 1995) and is connected to the limbic system, including the AMG. This link suggests a role of ACC in the appraisal and regulating of emotions (Etkin et al., 2011) and in encoding emotional significance of auditory stimuli.

### Future directions

Our study provides a clear example of how establishing body ownership in VR outside of the MRI environment, in combination with a 3D video during fMRI measurements, greatly extends the opportunities for adopting more naturalistic methodologies in social and affective neuro-imaging experiments. The scarcity of human neuroscience studies using dynamic threatening stimuli contrasts with the relevance of personal space intrusion in the perception of threat, both for the victims (Lloyd et al., 2006) and for the aggressors (Schienle et al., 2017). As static threatening stimuli may fail to evoke realistic responses, naturalistic immersive 3D motion stimuli appear better suited to modulate the approach of threatening stimuli within a naturalistic environment that the participants may perceive as real. Here, we used a single short stimulus preceded by two different types of VR exposure to isolate the effect of perspective, keep all visual stimulus properties equal and maximize the perceived realism and emotional impact with only one stimulus repetition. However, this does limit our findings to this specific stimulus and does not allow for further disentangling of neural responses to emotional versus neutral stimuli within nearby space. Future studies may further vary the types of stimuli used and test whether synchronization of activity in the fronto-parietal brain network is specific for emotional stimuli after 1PP exposure or applies to other types of stimuli as well. Another line of future study could relate to potential gender differences in body ownership and threat experience. In this study, we embodied all participants (male and female) in a female virtual character. For the female participants it may have been easier to place themselves in the victims’ shoes than for the male participants. Moreover, male and female participants may respond differently to a male aggressor (Wabnegger et al., 2016). Future work could involve the design of a study with a larger number of participants, as our study was underpowered to perform gender specific analyses, and a simpler design, which would allow for disentangling possible gender differences. Our study shows that 1PP visuo-motor exercises in VR can be used to prime subsequent experiences such that behavioral and neuronal responses are aligned with the virtual victim. Combining immersive VR exposure with neuroimaging methods could provide a basis for behavioral change during therapeutic treatment (Seinfeld et al., 2018).

## Synthesis

Reviewing Editor: Daniela Schiller, Mount Sinai School of Medicine Decisions are customarily a result of the Reviewing Editor and the peer reviewers coming together and discussing their recommendations until a consensus is reached. When revisions are invited, a fact-based synthesis statement explaining their decision and outlining what is needed to prepare a revision will be listed below. The following reviewer(s) agreed to reveal their identity: Daphne Holt.

The manuscript was accepted for publication after being re-reviewed by the two previous reviewers.
